# Dynamic Risk and Protective Factors of NSSI in Middle School Students: A Longitudinal Study

**DOI:** 10.62641/aep.v54i4.2086

**Published:** 2026-08-15

**Authors:** Xinwei Yu, Li Zhang, Ye Yu, Xuejian Su, Yipeng Su, Defa Mo, Yuwei Yang, Bo Liu, Lingyun Lai, Lifang Zhou, Xiaopeng Deng

**Affiliations:** ^1^Department of Clinical Medicine, Health Science Center of Yangtze University, 434000 Jingzhou, Hubei, China; ^2^Social Psychiatry Research Group, Mental Health Center of Yangtze University, 434000 Jingzhou, Hubei, China; ^3^Department of Psychiatry, Jingzhou Mental Health Center, 434000 Jingzhou, Hubei, China; ^4^Department of Psychiatry, Jingzhou Rongjun Special Care Hospital, 434000 Jingzhou, Hubei, China

**Keywords:** middle school students, psychological problems, non-suicidal self-injury, longitudinal study

## Abstract

**Background:**

This study aimed to use longitudinal data from 2021 to 2023 to systematically examine the risk and protective factors and mechanisms associated with non-suicidal self-injury (NSSI) among middle school students in Jingzhou City, and to provide empirical evidence for targeted school-based psychological interventions.

**Methods:**

A baseline survey was conducted in 2021 (T1) among 652 first-year junior high school students from two schools in Jingzhou City. In 2023 (T2), 585 participants were successfully followed up (follow-up rate: 89.72%). Participants were categorized into four groups: no NSSI, incident NSSI, NSSI remission, and persistent NSSI. Intergroup differences in characteristics were analyzed using chi-square tests, Kruskal–Wallis H tests, and post hoc comparisons. Three binary logistic regression models were constructed to explore the predictive effects of baseline (T1) factors on incident, remitted, and persistent NSSI, respectively. The significance level was set at α = 0.05.

**Results:**

The overall rate of NSSI decreased markedly from 22.05% to 8.55% over the two-year period. Multivariable analysis showed that in the model predicting NSSI remission (no NSSI group vs. remission group), individuals whose depressive symptoms decreased were significantly more likely to have discontinued NSSI (adjusted odds ratio (aOR) = 8.43, 95% confidence interval (CI): 3.01–23.60). Those with stable anxiety symptoms had a significantly higher rate of NSSI remission compared with those whose anxiety worsened (aOR = 0.35, 95% CI: 0.18–0.67). Gender-stratified analyses showed that among boys, a reduction in internet addiction was a significantly protective factor for NSSI remission (aOR = 8.87, 95% CI: 1.81–43.48). Among girls, both a decrease in depressive symptoms (aOR = 18.53, 95% CI: 3.36–102.26) and stable anxiety symptoms (aOR = 0.31, 95% CI: 0.14–0.72) were significantly associated with NSSI remission.

**Conclusions:**

NSSI behaviors among middle school students exhibit a high remission rate (82.17%) over time. The findings underscore the close association between NSSI and dynamic psychological problems. Prevention and intervention strategies of NSSI should be developed according to the longitudinal follow-up study, such as specific interventions of internet use behaviors for males and depressive and anxiety symptoms for females.

## Introduction

Non-suicidal self-injury (NSSI) refers to the deliberate infliction of physical harm to oneself without suicidal intent [1]. Recent research has highlighted the alarming prevalence of NSSI among adolescents worldwide, with a global detection rate of 17.20% [2] and a rate of 24.70% in China [3]. Notably, in China, the prevalence of NSSI among females is 1.35 times higher than that among males [4]. Although NSSI itself is rarely lethal, it is closely linked to clinical functional impairment and serves as a critical early warning sign for suicide attempts and behaviors [5]. Evidence suggests that NSSI is an independent risk factor for both suicide attempts and completed suicide. According to the Oxford University Suicide Research Center, individuals with NSSI have a 0.70% risk of suicide within the first year-66 times higher than that of the general population, and this risk escalates to 1.70%, 2.40%, and 3.00% at the 5, 10, and 15 year marks, respectively [6]. In summary, adolescent NSSI has emerged as a global public health challenge. Its high detection rate and upward trend demand proactive and effective interventions to protect the physical and mental health of adolescents.

This study focuses on a longitudinal investigation of Chinese middle school students. Early adolescence (ages 10–14) is a critical period characterized by diminished emotional regulation abilities, a sharp increase in academic pressure, and social role reconstruction. These developmental features make this age group particularly vulnerable to NSSI [7, 8]. Specifically, middle school students are transitioning from elementary to secondary education. During this period, they must adapt to higher academic demands while simultaneously navigating restructured interpersonal relationships and self-identity exploration. Such transitional pressures may lead them to resort to NSSI as a maladaptive coping mechanism during emotional dysregulation [9]. Moreover, existing literature indicates that early adolescence is a peak period for NSSI onset, with pronounced gender differences observed during this stage [10, 11, 12]. Therefore, understanding the trajectory changes of NSSI among Chinese middle school students between T1 and T2 is important for development of interventions for NSSI.

Moreover, there are limitations in previous studies of NSSI. First, there is a paucity of longitudinal follow-up studies on adolescent NSSI within the specific cultural context of China, particularly concerning the relationship between psychological changes and NSSI. Second, there is a lack of research focusing on gender and specific developmental trajectories of NSSI among Chinese middle school students. Thus, this study, using a longitudinal follow-up investigation, aims to explore the characteristics of NSSI behaviors among Chinese middle school students from T1 to T2. This study will investigate the developmental trajectories and predictive factors of NSSI, improve the understanding of NSSI during early adolescence, and provide robust empirical evidence for the formulation of early prevention and intervention measures.

## Materials and Methods

### Study Subjects

This study is a two-year follow-up investigation. The sample size was estimated based on the 10 events per variable (EPV) rule for binary outcome research [13], which requires at least 10 outcome events per predictor variable. Six key predictive variables were included. Referring to previous literature reporting an incidence of NSSI of approximately 24.70% [3], the minimum required number of outcome events was calculated to be 60. Accordingly, the minimum total sample size was estimated to be 219 participants. To account for potential attrition in longitudinal studies, the sample size was increased by 20%, resulting in a final target sample size of at least 274 participants.

This study was approved by the Ethics Committee of Jingzhou Mental Health Center (Approval No. 2021LL0501), and all procedures were conducted in accordance with the ethical principles of the Declaration of Helsinki [14]. A stratified cluster random sampling method was used to select the study population. In November 2021 (T1, baseline), after obtaining written informed consent from the participants and their legal guardians, a survey was conducted among all first year junior high school students from two schools in Jingzhou City. The same cohort was followed up in September 2023 (T2, follow-up). A total of 652 valid questionnaires were collected across the two waves of surveys. After excluding 67 invalid cases due to missing key demographic variables (e.g., age, sex) or core study variables, as well as those with excessively short response times (defined as an average response time of less than 2 seconds per question [15]), the final analytical sample comprised 585 participants. This sample size met the minimum requirement based on the EPV principle, and the valid response rate was 89.72%.

The participants’ ages ranged from 11 to 16 years, aligning with the age criteria for adolescents in developmental psychology [16]. The sample included 292 boys (49.91%). At T1, the mean age of the students was 12.2 ± 0.43 years. At T2, the mean age was 14.2 ± 0.48 years.

### Study Instruments

#### General Information Questionnaire

The study employed the “Sunshine Youth Mental Health Survey” [17] developed by Professor Mao-Sheng Ran of West China Hospital, Sichuan University. The questionnaire consists of two sections: general demographic information and items related to mental health. Demographic variables include age, grade, gender, and parental education level, and etc. Psychological stress was assessed on a 1 to 10 point Likert scale. Self‑injurious behavior was measured with the question, “In the past 12 months, have you engaged in any self‑harm behaviors?”. Response options were: “No”, “Once”, “2-4 times”, and “5 times or more”. In this study, any occurrence of self‑harm (≥1 episode) was classified as “presence of self injury behavior”, whereas no episodes (0) were classified as “absence of self injury behavior” [18].

#### Psychological Health Questionnaire

The assessment battery consisted of the following self report instruments: the Patient Health Questionnaire 9 (PHQ-9), the Generalized Anxiety Disorder 7 (GAD 7), the Insomnia Severity Index (ISI), and the Internet Addiction Test (IAT).

The Chinese version of the PHQ-9 has demonstrated good reliability and validity in both general and adolescent populations [19, 20]. This scale assesses depressive symptoms over the past two weeks, covering nine items: anhedonia, depressed mood, sleep disturbance, fatigue, appetite disturbance, guilt, concentration difficulties, psychomotor retardation or agitation, and suicidal or self-injury thoughts. Each item is rated on a 4-point Likert scale (0 = not at all, 1 = several days, 2 = more than half the days, 3 = nearly every day). A total score ≥ 5 indicates the presence of depressive symptoms, with higher scores reflecting greater severity of depression [21]. In this study, Cronbach’s α coefficients for this scale were 0.91 and 0.91 at T1 and T2, respectively.

The Chinese version of the GAD-7 has been validated in Chinese populations, showing satisfactory reliability and validity [22, 23, 24]. This scale assesses seven core anxiety symptoms over the past two weeks, including nervousness, uncontrollable worry, excessive worry, difficulty relaxing, restlessness, irritability, and fear. Each item is rated on a 4-point Likert scale (0 = not at all, 1 = several days, 2 = more than half the days, 3 = nearly every day). A total score ≥ 5 indicates the presence of anxiety symptoms, with higher scores indicating greater anxiety severity. In this study, Cronbach’s α coefficients for this scale were 0.93 and 0.93 at T1 and T2, respectively.

The Insomnia Severity Index (ISI) is a self-report instrument consisting of seven items used to assess insomnia severity. The evaluation dimensions include difficulty falling asleep, difficulty maintaining sleep, early morning awakening, satisfaction with sleep, interference with daytime functioning, noticeability of sleep problems by others, and distress caused by sleep difficulties. Each item is rated on a 5-point Likert scale, with total scores ranging from 0 to 28. A total score ≥ 8 indicates the presence of sleep problems, with higher scores reflecting greater insomnia severity [25]. The scale has been validated in Chinese adolescent populations, demonstrating sound psychometric properties [26, 27], with a Cronbach’s α coefficient of 0.83 and a two-week test–retest reliability of 0.79 [28]. In this study, Cronbach’s α coefficients for this scale were 0.88 and 0.87 at T1 and T2, respectively.

Internet use was assessed using the Internet Addiction Test (IAT) [29] developed by Young in 1996. This scale has been widely applied in both international and domestic research and has demonstrated satisfactory reliability and validity [30]. The scale consists of 20 items, each rated on a 5-point Likert scale (1 = rarely, 2 = occasionally, 3 = sometimes, 4 = often, 5 = always), with total scores ranging from 20 to 100. Higher scores indicate more severe internet addiction tendency [29]. Scores of 20–49 are generally considered to reflect normal internet use, while scores ≥ 50 are classified as indicating risk of internet addiction [31, 32]. In this study, Cronbach’s α coefficients for this scale were 0.93 and 0.94 at T1 and T2, respectively.

### Statistical Analysis

All statistical analyses were performed using SPSS software version 27.0 (IBM Corporation, Armonk, NY, USA). The significance level was set at p < 0.05. Continuous variables were presented as median (interquartile range) [Median (IQR)], and categorical variables were presented as frequency and percentage [n (%)].

#### Sample Grouping and Description

Based on the NSSI reports at T1 and T2, participants were categorized into four groups: (1) No NSSI group: no NSSI at both T1 and T2; (2) NSSI-remission group: with NSSI at T1 but not at T2; (3) Incident NSSI group: no NSSI at T1 but with NSSI at T2; (4) Persistent NSSI group: with NSSI at both T1 and T2.

#### Between-Group Comparisons: Univariate Analysis

To compare demographic and clinical characteristics among the four participant groups, the following analytical approaches were employed. First, normality of all continuous variables was assessed using the Shapiro–Wilk test, and homogeneity of variances across groups was examined via Levene’s test. For categorical variables, overall group differences were evaluated using the chi-square (χ^2^) test. For continuous variables, given that most variables violated the assumptions of normal distribution or homogeneity of variances (*p* < 0.05), the non-parametric Kruskal–Wallis H test was uniformly applied for overall comparisons. When the omnibus test was statistically significant (*p* < 0.05), further post-hoc pairwise comparisons were conducted. For categorical variables, pairwise comparisons were performed using chi-square tests with Bonferroni correction (adjusted significance level: α’ = 0.05/number of comparisons). For continuous variables, Dunn’s test with Bonferroni adjustment was used to control for Type I error inflation due to multiple comparisons. Results of post-hoc comparisons are indicated in Table 1 using distinct superscript letters (^a,b,c^), where groups sharing the same superscript letter do not differ significantly.

**Table 1.** **Demographic characteristics of participants across different groups**.

Variables	No-NSSI group	NSSI-remission group	Incident NSSI group	Persistent NSSI group	Chi‑square value	*p* value
	(n = 429)	(n = 106)	(n = 27)	(n = 23)	/H value	
Gender (female) n (%)	193 (44.99%)^a^	66 (62.26%)^a,b^	13 (48.15%)^b^	20 (86.96%)^c^	23.29	<0.001
Ethnicity (Han Chinese) n (%)	418 (97.44%)	101 (95.28%)	26 (96.30%)	23 (100.00%)	2.16	0.540
Paternal education (college degree or above) n (%)	126 (29.37%)	37 (34.91%)	10 (37.04%)	7 (30.43%)	9.10	0.168
Maternal education (college degree or above) n (%)	145 (33.80%)	43 (40.57%)	13 (48.15%)	9 (39.13%)	7.15	0.307
Quarantined in T1 n (%)	24 (5.59%)	6 (5.66%)	3 (11.11%)	1 (4.35%)	1.52	0.678
Physical exercise in T1 n (%)	240 (55.94%)^a^	50 (47.17%)^a^	17 (62.96%)^a^	7 (30.43%)^a^	8.63	0.035
Physical exercise in T2 n (%)	358 (83.45%)	83 (78.30%)	20 (74.07%)	16 (69.57%)	4.97	0.174
Psychological stress score at T1, Median (IQR)	2.00 (1.00–3.00)	2.00 (1.00–4.00)	3.00 (1.00–5.00)	3.00 (1.00–5.00)	1.80	0.407
Psychological stress score at T2, Median (IQR)	3.00 (1.00–5.00)^a^	4.00 (2.00–5.00)^b^	6.00 (4.00–8.00)^c^	6.00 (4.00–7.00) ^c^	25.60	<0.001

Note: NSSI, non suicidal self injury. (a) Different superscript letters (^a,b,c^) indicate statistically significant differences between groups in post hoc pairwise comparisons (*p* < 0.05). Bonferroni adjusted exact chi square tests were used for categorical variables, and Dunn Bonferroni tests were used for continuous variables. Groups sharing the same letter do not differ significantly. (b) IQR = interquartile range (Q1–Q3).

#### Longitudinal Risk Factor Analysis: Prediction Models Based on Logistic Regression

To explore the longitudinal predictive effects of baseline (T1) factors on different NSSI outcome trajectories at follow-up (T2), three independent binary logistic regression models were constructed. First, Prediction Model for Incident NSSI: This model included the No NSSI group and the Incident NSSI group. The dependent variable was whether NSSI newly occurred at T2 (Incident NSSI group = 1, No NSSI group = 0). Second, Prediction Model for NSSI Remission: This model included the No NSSI group and the NSSI-remission group. The dependent variable was whether NSSI remitted at T2 (NSSI-remission group = 1, No NSSI group = 0). Third, Prediction Model for Persistent NSSI: This model included the No NSSI group and the Persistent NSSI group. The dependent variable was whether NSSI persisted at T2 (Persistent NSSI group = 1, No NSSI group = 0).

In all regression models, candidate predictors included demographic characteristics (e.g., gender) and mental health-related variables (e.g., anxiety, depression, insomnia, internet addiction, physical exercise willingness, and psychological stress scores) that showed significant differences in univariate analyses. The models used forward stepwise regression (based on the likelihood ratio, Forward LR) to select variables. This approach aimed to identify factors with independent predictive effects on the outcome variable from the candidate predictor set, while controlling for potential multicollinearity. Results were reported as adjusted odds ratios (aOR) with corresponding 95% confidence intervals (95% CI).

## Results

### Common Method Bias Assessment

Common method bias was evaluated using Harman’s single‑factor test. Exploratory factor analysis of the questionnaires from both years extracted 18 factors with eigenvalues greater than 1. The first factor accounted for 25.54% and 20.70% of the variance, respectively-both well below the conventional 40% threshold-indicating that the data were not substantially affected by common method bias.

### Sample Attrition and Baseline Characteristics

Comparisons between participants who were lost to follow-up and those who remained in the study revealed no significant differences in ethnicity (χ^2^ = 0.00, *p* = 0.971), gender (χ^2^ = 0.35, *p* = 0.554), current cohabitation status (χ^2^ = 1.20, *p* = 0.879), household income (χ^2^ = 3.05, *p* = 0.271), only child status (χ^2^ = 0.17, *p* = 0.683), paternal education level (χ^2^ = 4.46, *p* = 0.348), maternal education level (χ^2^ = 1.91, *p* = 0.752), weekly frequency of exercise (χ^2^ = 0.82, *p* = 0.844), duration of each exercise session (χ^2^ = 0.95, *p* = 0.813), occurrence of NSSI behavior (χ^2^ = 0.05, *p* = 0.829), IAT scores (Z = –1.93, *p* = 0.054), GAD 7 scores (Z = –0.69, *p* = 0.491), PHQ 9 scores (Z = –0.83, *p* = 0.408), ISI scores (Z = –1.62, p = 0.105), and perceived psychological stress scores (Z = –1.08, *p* = 0.279). These findings indicate that attrition was not systematic and that the remaining sample did not suffer from structural bias.

### Prevalence and Longitudinal Trajectory of NSSI Behavior

Based on NSSI trajectories, the number of participants in four subgroups was as followings: (1) No NSSI group (n = 429, 73.33%); (2) NSSI-remission group (n = 106, 18.12%); (3) Incident NSSI group (n = 27, 4.62%); (4) Persistent NSSI group (n = 23, 3.93%).

At T1, the overall detection rate of NSSI was 22.05% (129/585), with rates of 14.73% (43/292) in boys and 29.35% (86/293) in girls. By T2, the overall detection rate decreased significantly to 8.55% (50/585; χ^2^ = 41.16, *p* < 0.001), with rates dropping to 5.82% (17/292) in boys and 11.26% (33/293) in girls.

As shown in Table 1, significant differences in baseline characteristics were observed among the different NSSI trajectory groups. Statistically significant differences were found across the four groups in gender distribution (χ^2^ = 23.29, *p* < 0.001), psychological stress scores in 2023 (Kruskal–Wallis H = 25.60, *p* < 0.001), and reported physical exercise rates in 2021 (χ^2^ = 8.63, *p* = 0.035).

Post hoc pairwise comparisons further revealed (see superscripts in Table 1): Regarding gender, the proportion of females in the Persistent NSSI group (86.96%) was significantly higher than that in the No NSSI group (44.99%), the NSSI remission group (62.26%), and the Incident NSSI group (48.15%); the proportion of females in the NSSI remission group was also higher than that in the No NSSI group (all adjusted *p* < 0.05). For psychological stress scores at T2, the Persistent NSSI group (5.78) and the Incident NSSI group (5.96) did not differ significantly, but both groups had significantly higher psychological stress scores than the NSSI remission group (3.69) and the No NSSI group (2.94); the NSSI remission group also scored higher psychological stress than the No NSSI group. Although the overall test for physical exercise willingness at T1 was significant, none of the pairwise comparisons reached the adjusted significance level.

### Univariate Analysis of Factors Influencing the Occurrence of NSSI Behavior

**Fig. 1.**
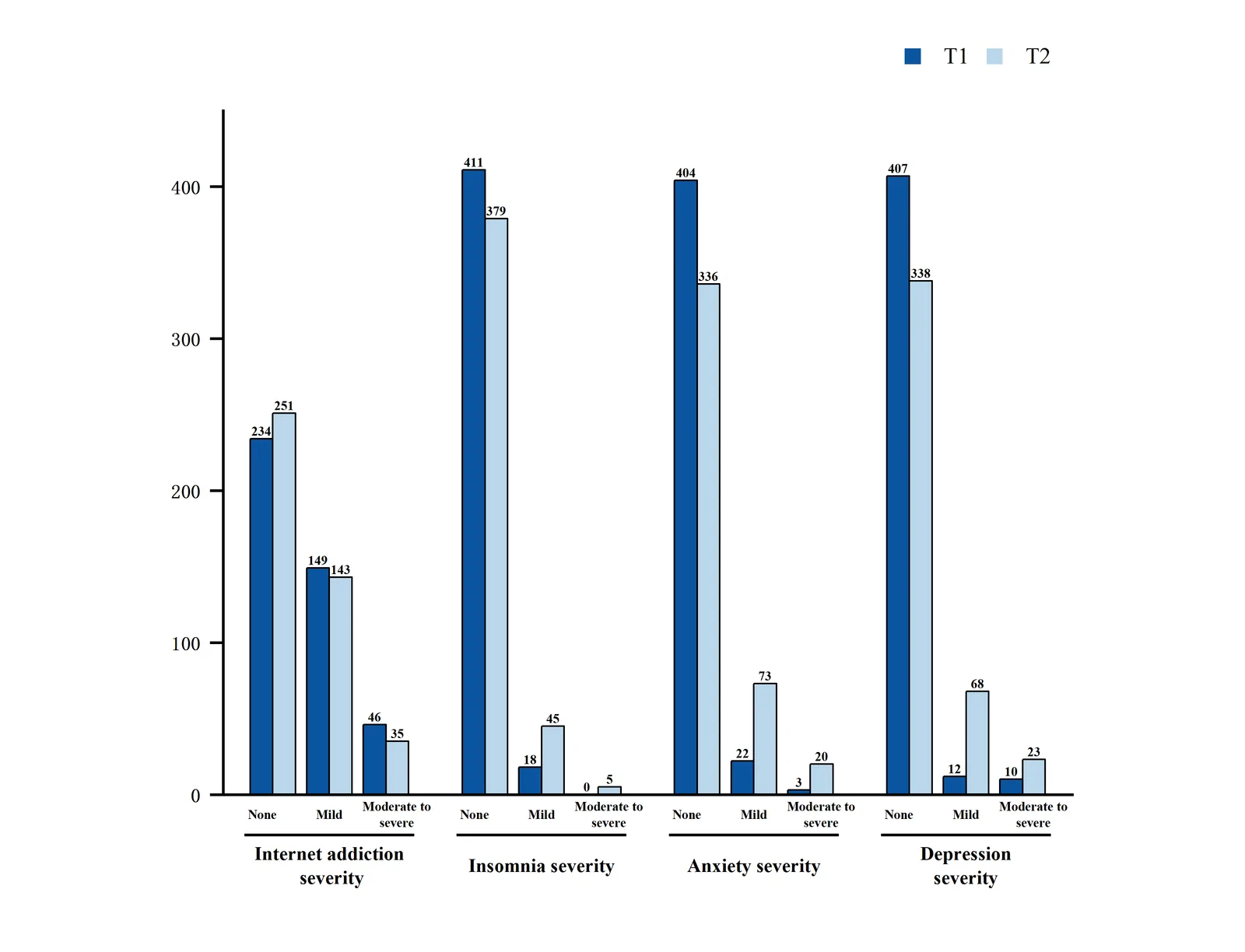
**Comparison of psychological status distribution between T1 and T2 in the non-NSSI group**.

**Fig. 2.**
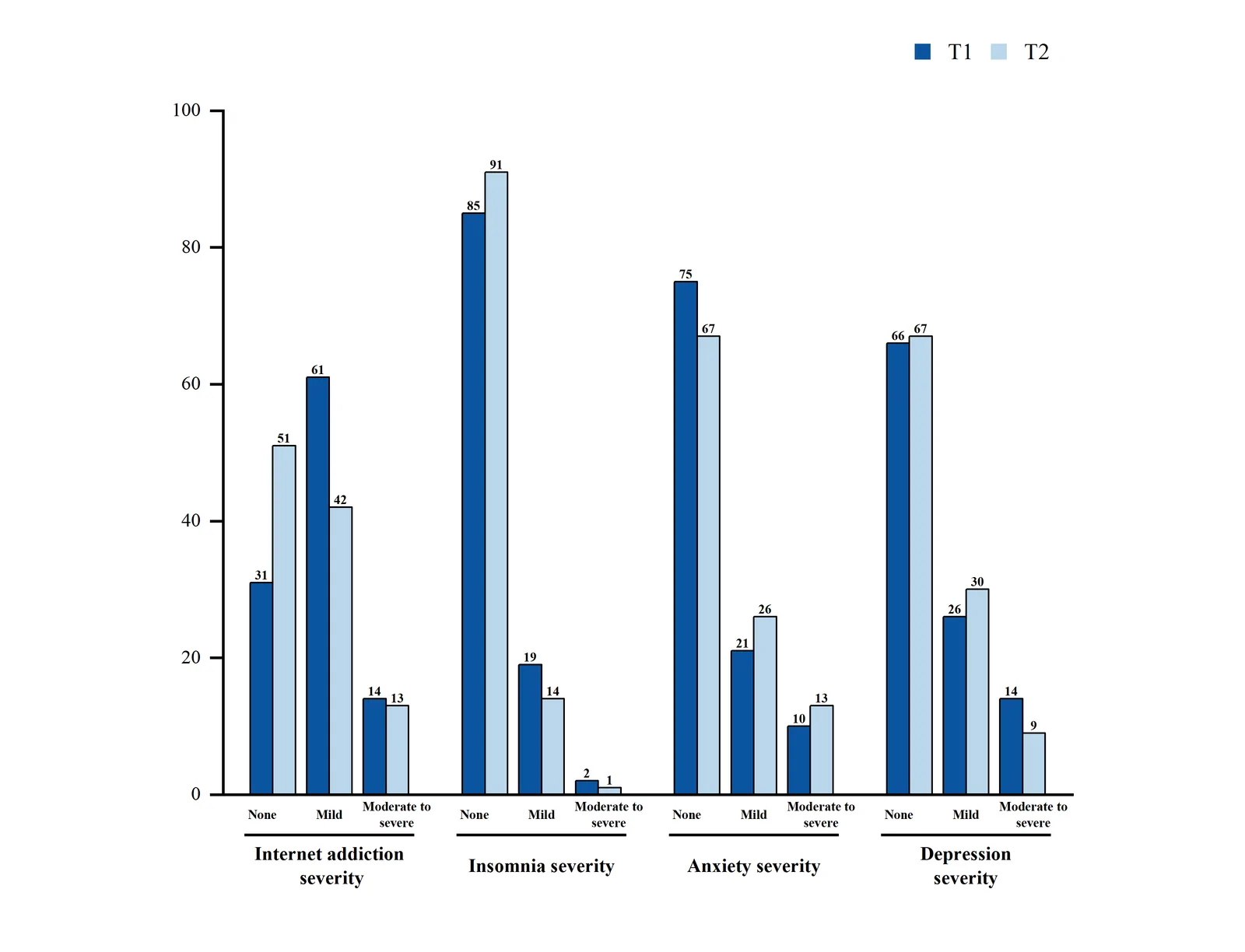
**Comparison of psychological status distribution at T1 and T2 for the NSSI-remission group**.

**Fig. 3.**
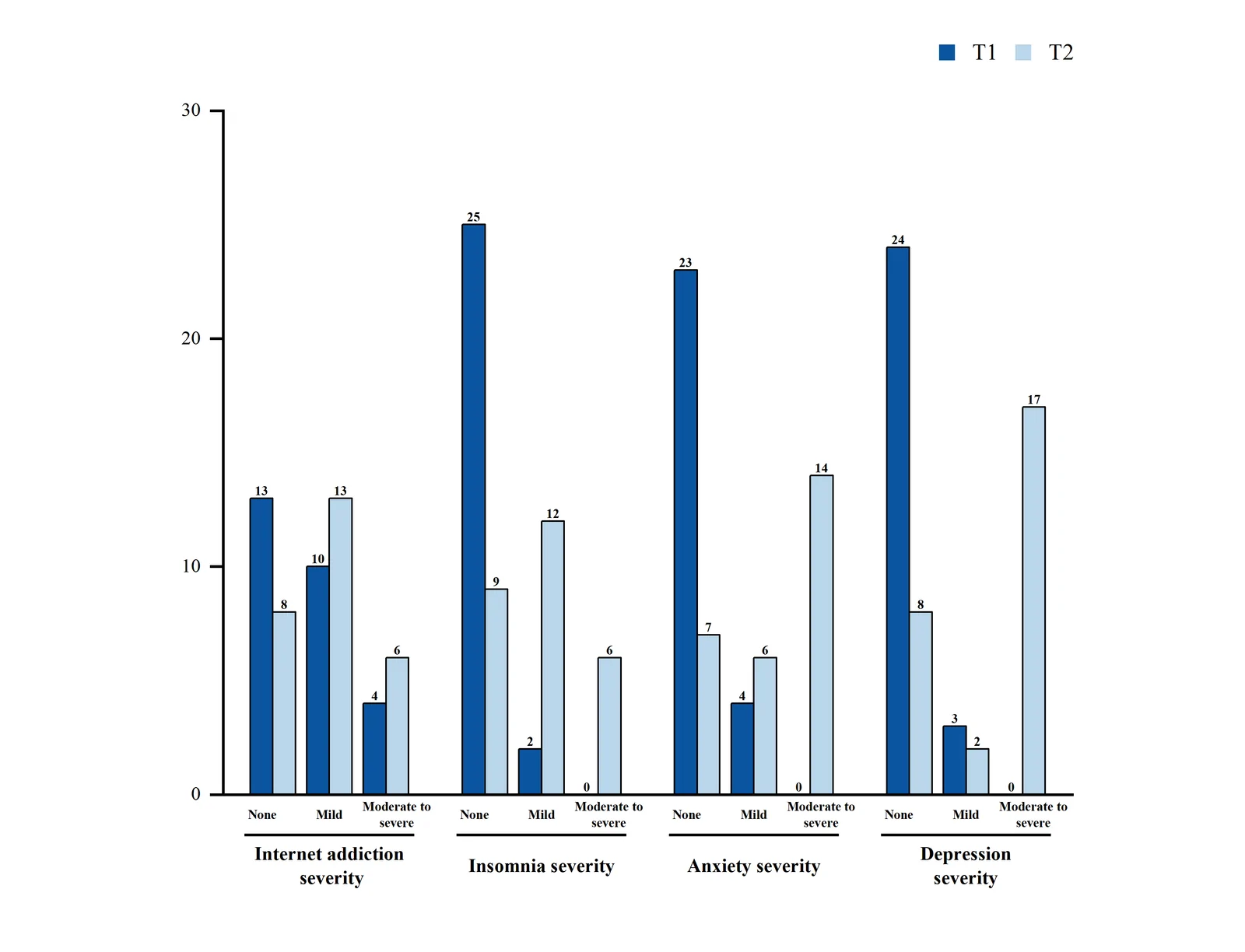
**Comparison of psychological status distribution at T1 and T2 for the incident NSSI group**.

**Fig. 4.**
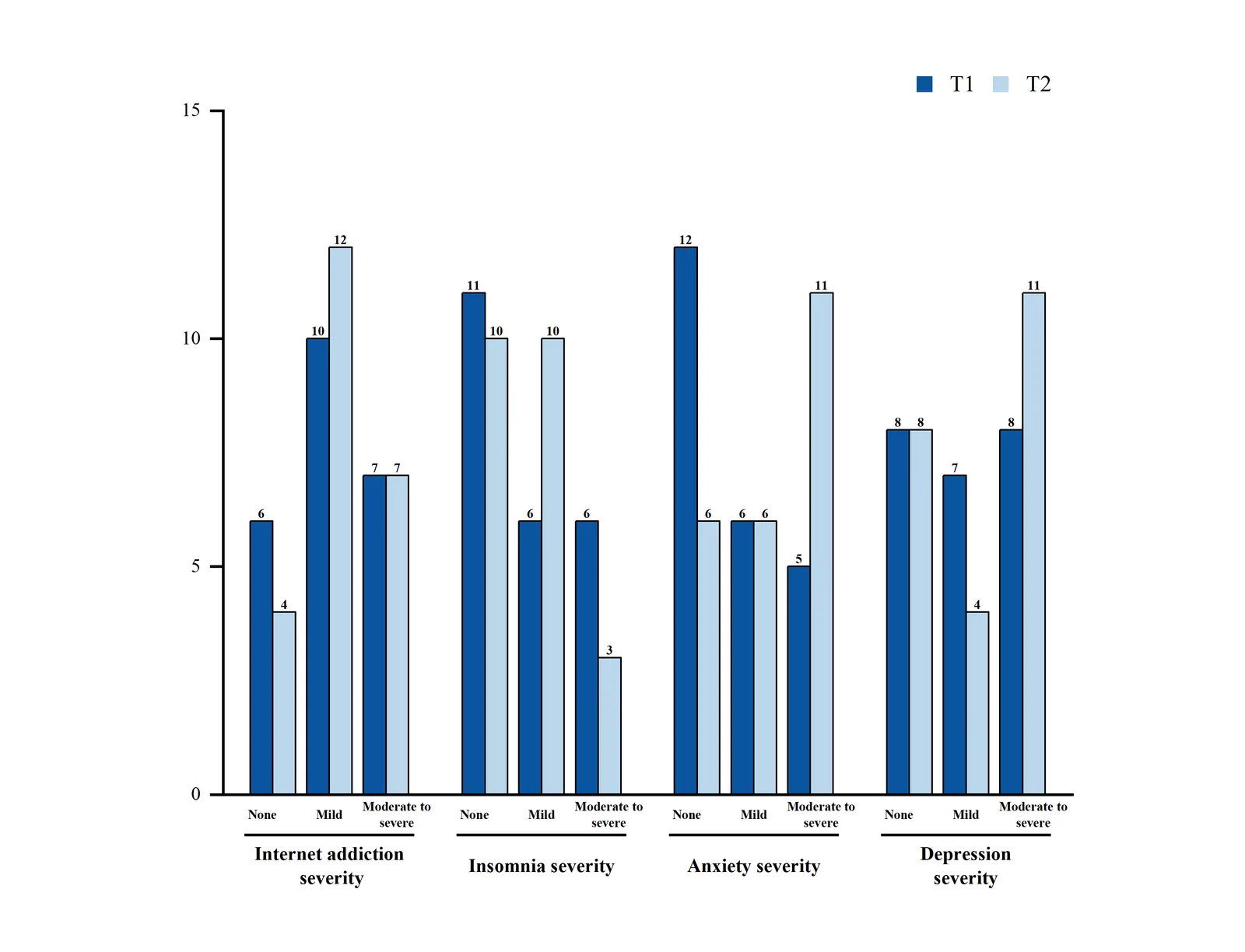
**Comparison of psychological status distribution at T1 and T2 for the persistent NSSI group**.

Univariate analyses revealed statistically significant associations between NSSI occurrence and several variables across the two time points. Specifically, participation in physical exercise at T1 (χ^2^ = 8.63, *p* = 0.035) and the severity of Internet addiction (IAT scores) at both T1 (χ^2^ = 35.37, *p* < 0.001) and T2 (χ^2^ = 31.00 , *p* < 0.001) showed significant associations with NSSI. Moreover, the severity of insomnia (ISI scores) at T1 (χ^2^ = 148.79, *p* < 0.001) and T2 (χ^2^ = 126.87, *p* < 0.001), anxiety (GAD-7 scores) at T1 (χ² = 89.41, *p* < 0.001) and T2 (χ^2^ = 133.13, *p* < 0.001), as well as depression (PHQ-9 scores) at T1 (χ^2^ = 163.30, *p* < 0.001) and T2 (χ^2^ = 158.27, *p* < 0.001) were also significantly associated with NSSI. The results for the association between sociodemographic and mental health variables and NSSI are presented in Table 1 and Figs. 1–4.

### Multivariate Logistic Regression Analysis of Factors Influencing the Occurrence of NSSI Behaviorv

The occurrence of NSSI behaviors was used as the dependent variable (0 = No, 1 = Yes). Independent variables included changes in the degree of internet addiction (1 = decreased, 2 = unchanged, 3 = increased), changes in psychological stress (1 = score decreased, 2 = score unchanged, 3 = score increased), changes in willingness to engage in physical exercise (1 = became negative, 2 = unchanged, 3 = became positive), changes in the severity of insomnia (1 = decreased, 2 = unchanged, 3 = increased), changes in the level of anxiety (1 = decreased, 2 = unchanged, 3 = increased), and changes in the level of depression (1 = decreased, 2 = unchanged, 3 = increased). For categorical variables, the ”increased” category was set as the reference group. Stepwise regression was employed.

In all binary logistic regression models in this section, the reference group for psychological status change variables (depression, anxiety, insomnia, internet addiction, psychological stress, and physical exercise willingness) was set as “worsened/increased severity.” Comparisons were conducted using the No-NSSI group (n = 429) as the reference, relative to the NSSI-remitted group (n = 106, primary analysis), the NSSI-incident group (n = 27, secondary analysis 1), and the NSSI-persistent group (n = 23, secondary analysis 2). An OR > 1 indicates that a given change direction (e.g., decreased, stable) is more likely to occur in the outcome group (e.g., remitted, incident, persistent) relative to the reference category of worsening; an OR < 1 indicates the opposite. All references to “predictors” or “associated factors” hereafter refer to statistical associations but not to causal direction.

Results of the Primary Analysis Group (No-NSSI group vs. Remitted group) showed that after controlling for other variables, a decrease in depressive symptoms was identified as a significant protective factor for the remission of NSSI behaviors (aOR = 8.43, 95% CI: 3.01–23.60). Additionally, stable anxiety levels were also associated with a reduced risk of NSSI (aOR = 0.35, 95% CI: 0.18–0.67). The detailed results are presented in Table 2.

**Table 2.** **Impact of Changes in Psychological Status on the Occurrence of NSSI Behaviors in the Primary Analysis Group**.

Variables		B	S.E.	aOR (95%CI)	*p* value
Changes in depression level					
	Decreased	2.13	0.53	8.43 (3.01–23.60)	<0.001
	Unchanged	0.32	0.36	1.38 (0.68–2.80)	0.379
Changes in anxiety level					
	Decreased	0.15	0.52	1.16 (0.42–3.18)	0.772
	Unchanged	–1.06	0.33	0.35 (0.18–0.67)	<0.001

Note: B, regression coefficient; S.E., standard error; aOR, adjusted odds ratio; CI, confidence interval.

Results of Secondary Analysis 1 (No-NSSI group vs. Incident group) showed that non deterioration of insomnia severity (aOR = 0.12, 95% CI: 0.04–0.32) and non deterioration of depressive level (aOR = 0.22, 95% CI: 0.08–0.65) were significant protective factors against the new onset of NSSI behaviors. Results of Secondary Analysis 2 (No-NSSI group vs. Persistent group) indicated that a reduction in psychological stress (aOR = 0.12, 95% CI: 0.02–0.63), a decrease in depressive symptoms (aOR = 4.98, 95% CI: 1.18–21.00), and non deterioration of depressive level (aOR = 0.23, 95% CI: 0.07–0.74) were protective factors against the persistence of NSSI behaviors.

Gender-stratified logistic regression analysis further revealed heterogeneity in influencing factors across sexes. Among males, a reduction in the internet dependence was identified as a key protective factor for the remission of NSSI behaviors (aOR = 8.87, 95% CI: 1.81–43.48). Among females, a decrease in depressive symptoms was strongly associated with behavioral remission (aOR = 18.53, 95% CI: 3.36–102.26), while stable anxiety levels also was a protective factor (aOR = 0.31, 95% CI: 0.14–0.72). Detailed results are presented in Tables 3 and 4.

**Table 3.** **Impact of changes in psychological status on the occurrence of NSSI behaviors in the male subgroup of the primary analysis group**.

Variables		B	S.E.	aOR (95%CI)	*p* value
Changes in depression level					
	Decreased	0.93	0.80	2.53 (0.52–12.19)	0.248
	Unchanged	–0.38	0.63	0.69 (0.20–2.33)	0.545
Changes in anxiety level					
	Decreased	1.23	0.85	3.41 (0.64–18.09)	0.149
	Unchanged	–0.35	0.62	0.71 (0.21–2.40)	0.578
Changes in the degree of internet addiction					
	Decreased	2.18	0.81	8.87 (1.81–43.48)	0.007
	Unchanged	1.36	0.80	3.91 (0.82–18.66)	0.087

Note: B, regression coefficient; S.E., standard error; aOR, adjusted odds ratio; CI, confidence interval.

**Table 4.** **Impact of changes in psychological status on the occurrence of NSSI behaviors in the female subgroup of the primary analysis group**.

Variables		B	S.E.	aOR (95%CI)	*p* value
Changes in depression level					
	Decreased	2.92	0.87	18.53 (3.36–102.26)	<0.001
	Unchanged	0.42	0.46	1.53 (0.62–3.75)	0.358
Changes in anxiety level					
	Decreased	–0.37	0.77	0.69 (0.15–3.15)	0.634
	Unchanged	–1.16	0.42	0.31 (0.14–0.72)	0.006

Note: B, regression coefficient; S.E., standard error; aOR, adjusted odds ratio; CI, confidence interval.

Results indicated that among males, non-deterioration of anxiety levels was a protective factor against the new onset of NSSI (aOR = 0.07, 95% CI: 0.02–0.24). Among females, non-deterioration of insomnia severity was a protective factor against the new onset of NSSI (aOR = 0.02, 95% CI: 0.00–0.10). Other significant protective factors were identified only among females, including a reduction in psychological stress (aOR = 0.06, 95% CI: 0.01–0.64), non-deterioration of insomnia severity (aOR = 0.23, 95% CI: 0.06–0.92), and non-deterioration of depressive levels (aOR = 0.29, 95% CI: 0.08–0.99).

## Discussion

This study is a two-year follow-up survey (from 2021 to 2023) involving 585 middle school students in China to dynamically investigate the evolving trajectories of NSSI behaviors. It further explores the associations between changes in multiple psychological risk factors and the risk of NSSI occurrence, with a specific focus on the differential roles of gender in these relationships.

First, the results of this study showed that the overall detection rate of NSSI among adolescents significantly decreased from 22.05% at T1 to 8.55% at T2. This declining trend aligns with findings of several longitudinal studies suggesting that the incidence of NSSI may naturally decline with age and the maturation of neurocognitive functions, such as prefrontal cortex development [33, 34]. However, this overall decreasing trend in this study must be interpreted cautiously within its specific context. The follow-up period of this study coincided with the transition from the peak of the COVID-19 pandemic (2021) to a phase of gradual resumption of social activities (2023). Existing research indicates that social isolation, pressures of online learning, and uncertainty during the pandemic may have temporarily elevated the risk of NSSI [13, 35]. Therefore, the observed decline in NSSI may partially reflect a “post-pandemic recovery” effect, wherein the resumption of normal school life and social activities may alleviate certain psychological stressors for some adolescents [36, 37]. The results of this study also showed that while psychological stress, anxiety, and depressive symptoms fluctuated across groups from T1 to T2, some indicators showed improvement.

This study identified subgroups with distinct trajectories of NSSI including No NSSI, NSSI Remission, Incident NSSI, and Persistent NSSI groups. This classification highlights the heterogeneity of NSSI risk, indicating that a notable proportion of individuals remain at risk for either incident or persistent NSSI (collectively 8.55%) even against a generally optimistic background. In particular, the presence of a Persistent NSSI group (3.93%) suggests that some adolescents may develop a stable and difficult-to-change pattern of self-injurious behavior, which aligns with the perspective of the Four-Function Model, wherein NSSI is viewed as a learned, automatic emotion regulation or self-punishment strategy [18, 38]. Early identification and long-term intervention for this high-risk subgroup are crucial.

The core finding of this study lies in the observation that the dynamic change patterns of psychological states are more predictive of NSSI outcomes than their static levels. In the primary analysis (No NSSI group vs. NSSI-remission group), we found that individuals whose depressive symptoms decreased were approximately eight times more likely to discontinue NSSI compared to those whose depressive symptoms worsened. This finding has significant clinical and theoretical implications. It suggests that for adolescents already engaging in NSSI, improvement in depressive symptoms serves as a strong signal for NSSI remission. This supports the central role of interventions targeting comorbid depression in the treatment of NSSI [39, 40]. Furthermore, maintaining stable anxiety symptoms (compared to worsening) was also associated with a higher likelihood of NSSI remission. This may indicate that during adolescence, preventing further deterioration in anxiety levels-even without significant reduction - could create a relatively stable emotional environment conducive to ceasing NSSI behavior. Stable, non-worsening anxiety levels might be linked to a degree of emotional alertness and help-seeking willingness, preventing complete emotional dysregulation [41].

However, it is noteworthy that in the secondary analysis (No NSSI group vs. Persistent NSSI group), a decrease in depressive symptoms also predicted a higher risk of persistent NSSI (aOR = 4.98). This may reveal heterogeneous underlying psychological mechanisms across different trajectory subgroups. For individuals in the Persistent NSSI group, NSSI may have evolved into an ingrained coping habit or a component of self-identity that operates independently of specific depressive mood states. Even if scores on depression symptom scales show improvement, core deficits in emotion regulation, self-critical cognitions, or interpersonal communication functions may remain unchanged, thereby sustaining NSSI behavior. This finding caution that focusing solely on the reduction of depression symptom scale scores may be insufficient to predict NSSI behaviors for all individuals. It underscores the need for comprehensive assessment of NSSI, behavioral frequency, and cognitive patterns.

This study has also revealed the significant gender differences in NSSI risk pathways, which provides an empirical basis for gender-specific prevention strategies. For males, a reduction in the severity of internet addiction was the strongest factor predicting NSSI remission (aOR = 8.87). This finding may support the ”functional substitution” hypothesis [42, 43]. Internet use, particularly immersive gaming or social media engagement, is often employed by adolescents, especially males, as a means to escape real-world stress and regulate negative emotions. When such overt escapist behavior (e.g., internet addiction) is reduced, whether forced or voluntary, and if the individual fails to develop other effective emotion-regulation skills, accumulated psychological distress may instead be expressed or released through another externalizing behavior - NSSI. Therefore, for males with tendencies toward excessive internet use, intervention must extend beyond behavioral control to include concurrent training in emotion identification, stress management, and adaptive coping skills. This is essential to fill the resulting “behavioral vacuum” and prevent risk displacement [44].

For females, the results of this study indicate the risk of NSSI with stronger “internalizing” features. The association between decreased depressive symptoms and NSSI remission in females was significantly stronger than that in males (aOR = 18.53 vs. non-significant in males). This highlights the central role of depressive mood in the pathological mechanisms of NSSI among females [39]. Females may be more prone to internalizing psychological distress, with NSSI often used to regulate intense, ineffable depressive emotions or feelings of self-loathing [45]. Concurrently, stable anxiety symptoms also demonstrated a clear protective effect in females. This suggests that preventing an escalation of anxiety is particularly crucial for disrupting the pathway from emotional distress to self-injurious behavior in females [40, 46]. Furthermore, in the analyses for incident and persistent NSSI among females, a worsening of insomnia was also identified as a risk factor, indicating that sleep disturbances may serve as an important precursor of NSSI.

It should be noted that this study had a few limitations. First, this study employed a single self report item to measure NSSI behavior. While this method facilitates large scale epidemiological screening, it is unable to assess important dimensions of self injury, such as frequency, specific methods, severity, and functional intent, thereby limiting a comprehensive reflection of the complex clinical characteristics of NSSI. Additionally, the self report method may be influenced by recall bias and social desirability effects, further restricting the accuracy of the data. Future research could consider using more comprehensive and standardized assessment tools [47]. Moreover, combining multiple sources of information - such as clinical interviews and behavioral records - could help mitigate the potential biases associated with self report methods. Second, this study did not systematically measure and incorporate covariates directly related to the effects of the pandemic (e.g., duration of lockdowns, intensity of online learning, fear of infection, or changes in family economic pressure) on NSSI. Future research should integrate such macro- and micro-level pandemic-related factors into analytical models. Third, the sample was recruited from a single city (Jingzhou) in China, which limits the generalization of the results in this study to other areas of China.

## Conclusions

In summary, this longitudinal follow-up study reveals complex and gender specific associations between NSSI risk and patterns of change in psychological status in adolescents. These findings highlight the importance of focusing on the dynamic evolution of psychological symptoms rather than static levels in the prevention and intervention of NSSI. The results of this study also suggest the need for gender specific intervention strategies: for males, attention should be given to the emotional regulation gap following reduced internet dependence; for females, interventions should address depressive emotions and related harmful cognitive behavioral patterns. Future research should explore further on incorporating broader contextual factors-including the impact of major public health events such as the COVID-19 pandemic, employing more precise measures of NSSI, and testing the risk models in more diverse samples to facilitate the development of more effective and targeted prevention and intervention approaches.

## Availability of Data and Materials

The datasets generated and analyzed during the current study are not publicly available because they contain student information that they did not consent to have shared publicly at the individual level but aspects of the data set may be available from the corresponding authors on reasonable request.
